# Cerebellar compensation: a case of aphasia due to cerebellar hemorrhage

**DOI:** 10.1007/s00415-024-12276-6

**Published:** 2024-03-04

**Authors:** Yukiko Kinoshita, Masahiro Hatakeyama, Mika Otsuki, Takanobu Ishiguro, Etsuji Saji, Masato Kanazawa, Osamu Onodera

**Affiliations:** 1 Department of Neurology, Brain Research Institute, Niigata University, 1-757 Asahimachidori, Chuo-ku, Niigata City, Niigata 951-8585 Japan; 2 Faculty of Health Sciences, Hokkaido University, Kita 12, Nishi 5, Kita-ku, Sapporo, Hokkaido 060-0812 Japan

Dear Sirs,

In recent years, it has become evident that the cerebellum is involved not only in movement coordination but also in cognitive and behavioral functions and cognitive impairments due to cerebellar lesion are known as cerebellar cognitive affective syndrome (CCAS) [1]. CCAS is characterized by deficits in executive function, visuospatial cognition, affect regulation, and language [1, 2]; however, language impairments in CCAS are generally subtle and are only identified through special examinations.

Cases of aphasia caused by cerebellar stroke have been reported [3]; however, the underlying mechanisms of cerebellar-induced aphasia remain unknown. Inatomi et al. reported a patient with infratentorial infarction who developed aphasia after recovering from a speech disturbance due to left parietal lobe infarction. They hypothesized that disruption of the compensatory intensification of the crossed cerebellocerebral pathway caused by the initial infarction may be associated with infratentorial stroke-induced aphasia [4]. However, detailed information about the symptoms of speech disturbance due to the initial infarction was not provided in the report. Thus, it is unclear whether infratentorial stroke-induced aphasia was caused by the disruption of cerebellar compensation. Here, we report a case of a patient with cerebellar hemorrhage, in whom the same symptoms recurred after recovery from aphasia due to left inferior parietal lobule infarction. Our findings suggest the aphasia occurred due to the disruption of cerebellar compensation after left parietal lobe damage.

The patient was a 77-year-old right-handed Japanese woman with a twelfth grade education who had a history of hypertension, atrial fibrillation, and Sjogren's syndrome, and was administered oral edoxavan. She was admitted to a nearby hospital because of heart failure. During hospitalization, she had a cerebral infarction and presented aphasia characterized by phonemic paraphasia with repeated self-corrections (“*conduit d’approche*”) (e.g., she would try to rephrase “*mizu wo nomu*” ‘drink water’ as “*mizu wo mimu… mizumowo… mimo*”) and word-finding difficulties, whereas apraxia of speech and word comprehension deficits were not observed. Brain magnetic resonance imaging (MRI) showed cerebral infarction that extended from her left supramarginal gyrus to the anterior angular gyrus (Fig. 1a, b). After discharge, her symptoms gradually improved, and she had no difficulty in her daily life.

**Fig. 1:**
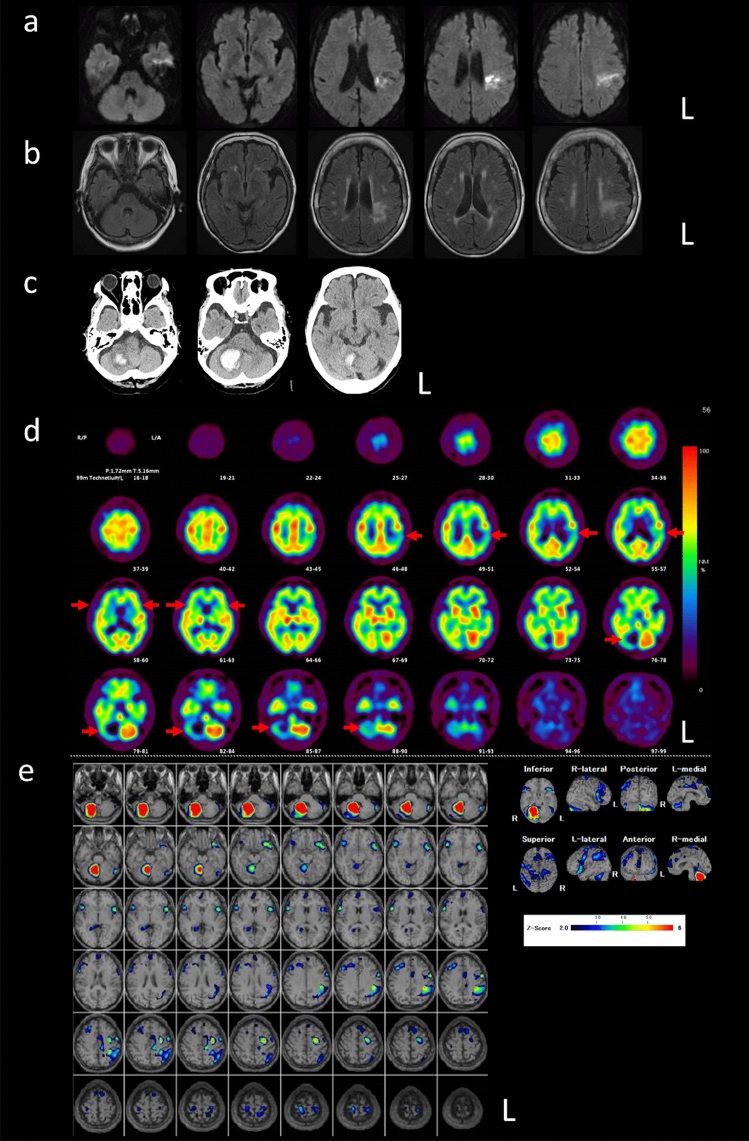
**a**, **b** Magnetic resonance imaging on the initial admission. Diffusion-weighted imaging (**a**) and fluid-attenuated inversion recovery imaging (**b**) showed a cerebral infarction extending from the left supramarginal gyrus to the anterior part of the angular gyrus. **c** Brain computed tomography on the second admission showed a hemorrhage in the right cerebellar hemisphere. **d **Single photon emission tomography (SPECT) with technetium-ethyl cysteinate dimer (^99^Tc-ECD) taken 23 days after the second admission revealed decreased cerebral blood flow (CBF) in the bilateral lateral frontal lobes, in addition to the right cerebellar hemisphere and the left inferior parietal lobule (arrows). Decreased CBF in the bilateral ACA territory was also observed. **e** SPECT data are analyzed with the Easy Z-score imaging system, and hypoperfusion with a Z-score > 2 is shown in color

One year after the onset of the cerebral infarction, the patient was transferred to our hospital via an ambulance. Upon neurological examination, the patient was alert, cooperative, and mentally stable; however, object naming was impaired. Furthermore, left-sided nystagmus and decomposition of her right upper limb were observed. Her muscle strength and superficial sensation were normal. A computed tomography (CT) scan of her brain revealed a hemorrhage in the right cerebellar hemisphere (Fig. 1c). Notably, her brain MRI showed no recent infarction.

Detailed neuropsychological evaluations were performed five days after admission. The patient’s speech contained irregularly distorted articulations, phonemic paraphasia, and neologism (e.g., she mistook her work for “*Yusetsu*” “*Obai*”). Her auditory verbal comprehension was impaired at the single-word level, and in the repetition task, phonemic paraphasia with repeated self-corrections (e.g., she mistook “*mado*” ‘window’ for “ka-bo” “ka-do”) was observed. In the object naming task, word-finding difficulties, phonemic paraphasia, and neologisms (e.g., she mistook “*keito*” ‘yarn’ for “*bo-ru*” or “*de-ten*”) were noted.

Sixteen days after admission, the patient incidentally developed infarctions localized in the territory of the bilateral anterior cerebral artery (ACA). Twenty-three days after admission, single-photon emission tomography (SPECT) with technetium-ethyl cysteinate dimer (^99^Tc-ECD) revealed decreased cerebral blood flow (CBF) in the bilateral lateral frontal lobes, right cerebellar hemisphere, and left inferior parietal lobule. Decreased CBF in the bilateral ACA territory was also observed, reflecting the presence of a new infarct (Fig. 1d, e).

Initially, the patient presented with phonemic paraphasia, with repeated self-corrections and word-finding difficulties due to an infarction of her left inferior parietal lobule. Although the aphasia symptoms improved, a recent cerebellar hemorrhage caused a recurrence of the phonemic paraphasia with repeated self-corrections and word-finding difficulty. Furthermore, auditory comprehension impairment, which had not been observed before the cerebellar hemorrhage, also developed.

Phonemic paraphasia with repeated self-correction is characteristic of conduction aphasia. Typical conduction aphasia lesions are found in the left supramarginal gyrus, left lower postcentral gyrus, and left insula [5]. In addition, phonemic paraphasia due to cerebellar damage has not been reported, except in one case in which Inatomi et al. assumed the disruption of cerebellar compensation [4]. Thus, we hypothesized that phonemic paraphasia was caused by the failure of this compensatory function and not by cerebellar damage alone. In the physiological state, the right cerebellar hemisphere is functionally connected with the left frontal and left parietal lobes, both of which are responsible for language function [6, 7]. In addition, transcranial direct current stimulation to the right cerebellar hemisphere has been reported to improve language function in both healthy adults and patients with aphasia [8, 9]. These results suggest a potential compensatory capacity of the cerebellum against aphasia due to damage to the left parietal lobe.

Auditory comprehension impairment is usually caused by lesions in the left temporoparietal and lateral frontal lobe [10, 11], and comprehensive impairment caused by cerebellar lesions has rarely been reported [12, 13]. Although the underlying mechanisms of comprehension impairment caused by cerebellar lesions remain unclear, one hypothesis suggests that it is caused by functional cerebral impairment due to crossed cerebellocerebral diaschisis [12]. In our case, SPECT revealed decreased CBF in the left lateral frontal lobe; therefore, dysfunction of this region caused by cerebellar lesions may be responsible for the comprehension impairment.

 The decreased CBF in the right frontal lobe including the right insular cortex was also observed in this case. The right cerebellum has functional connectivity to the right insular cortex [14]. Therefore, diaschisis of the right insular cortex due to the right cerebellar hemorrhage may result in the decreased CBF in the right insular cortex. The right insular cortex is reported to be associated with attention, salience processing, and syntactic processing [15, 16]; however, due to severe aphasia, we were unable to conduct detailed evaluation of such function in the patient. Thus, it is imperative that future research investigates whether right insular symptoms occur due to the right cerebellum damage in patients without aphasia.

## Data Availability

The data that support the findings of this study are available from the corresponding author, MH, upon reasonable request.
